# A Study on the Red Clay Binder Stabilized with a Polymer Aqueous Solution

**DOI:** 10.3390/polym13010054

**Published:** 2020-12-25

**Authors:** Jinsung Kim, Hyeonggil Choi, Hyeun-Min Rye, Keun-Byoung Yoon, Dong-Eun Lee

**Affiliations:** 1 School of Architecture, Civil Environment and Energy Engineering, Kyungpook National University, Daegu 41566, Korea; kjs07406@knu.ac.kr; 2 Department of Polymer Science and Engineering, Kyungpook National University, Daegu 41566, Korea; bc1532@knu.ac.kr (H.-M.R.); kbyoon@knu.ac.kr (K.-B.Y.)

**Keywords:** rammed-earth construction, polymer aqueous solution, poly(AA-*co*-AM), hydrogen bonding, microstructure

## Abstract

In this study, the performance evaluation was performed by adding a polymer aqueous (PA) solution as a new additive of the red clay binder for use in the rammed-earth construction method. The evaluation items were compressive strength, water erosion, shrinkage, crystal structure, and microstructure. As a result of the experiment, the binder was improved by efficiently bonding the silica particles by the polymerized polymer. It was confirmed that adding a PA solution to red clay enhances the compressive strength, which is further improved when 5 wt% poly(Acrylic acid(AA)-*co*-Acrylamide(AM)) is added to the PA solution. Microstructural analysis indicated that the addition of a PA solution facilitates effective bonding of the silica particles of red clay to form hydrogen bonding with poly(AA-*co*-AM) and encourages aggregate formation. Therefore, the study confirmed that PA solution can be applied to satisfy the performance requirements of the rammed-earth construction by improving the durability and strength of the binder.

## 1. Introduction

Rammed-earth construction (REC) is the most primitive soil-wall building method, and it uses an integral formwork made of wood or steel. An integral wall or floor is built by compacting red clay into the formwork using a 7–10 kg compactor [1,2,3]. This method can be used to form bearing walls with sufficient compaction. It manifests the natural texture of red clay in an interior design and exhibits the efficacy of natural red clay [4]. To obtain the desirable properties of REC, the ACP-EEC (Africa Caribbean Pacific-European Economic Community) and the New Mexico Adobe and Rammed Earth Building Code recommend 2.4 MPa and 200–300 psi (1.38–2.07 MPa) after curing for 28 days as the appropriate compressive strength for rammed-earth walls, respectively [5,6].

The materials used in REC are the ingredients of natural red clay. The strength of the red clay binder is lower than that of cement composites (concrete). The use of red clay to build structures has certain disadvantages; one such disadvantage is the cracking caused by shrinkage owing to the pores. To combat this disadvantage, lime or cement may be added as a solidifying agent [7,8,9,10,11,12]. They may develop long-term strength as carbonation occurs over a long period owing to pozzolanic reaction with the silica particles of red clay [9,13,14,15]. Limitations of using lime are related to the reaction process and space for hydration. Cement production has engineering benefits. However, it causes environmental pollution owing to the large volume of dust and CO_2_ emission [2]. Because the excessive addition of cement degrades the eco-friendliness of red clay, the amount of cement should be minimized as much as possible when it is used as a solidifying agent. For this purpose, existing studies have recommended approximately 8–12% as the appropriate cement content for securing the strength of rammed-earth walls [4,16]. Several studies have improved the performance of the red clay binder by manipulating the content of lime or cement; most of them have investigated the compressive strength of the binder. Certainly, cracking owing to moisture evaporation contributes to the durability and the shrinkage of the red clay binder and is not fully understood. It would be desirable to develop new soil stabilizers that can replace lime or cement, thereby reducing environmental pollution and promoting constructability.

Existing studies have investigated the moisture resistance of a red clay binder to improve its strength and durability. The moisture resistance issue is acute when the binder is cured outdoors through natural drying. Some studies have proposed polymers as stabilizers to address the issues involved in the strength and moisture resistance [17,18]. The compressive strength and durability of a red clay binder mixed with a polymer aqueous (PA) solution stabilizer have been compared to those of the conventional red clay binder. However, there exists a research gap between theory and practicability.

**Figure 1:**
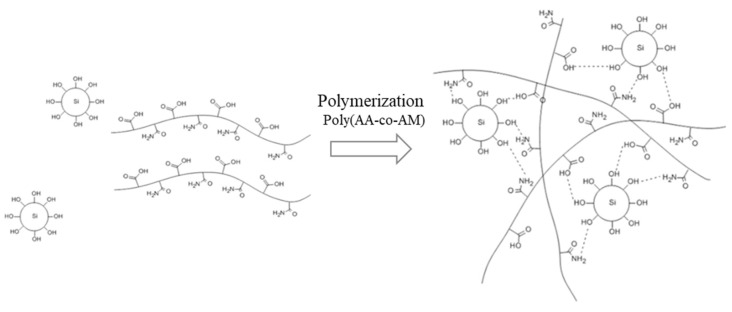
Reaction mechanism of the polymer aqueous solution stabilizer.

The reaction mechanism of the PA solution stabilizer used in this study is shown in Figure 1. PA solution used in this study was developed by professor Yoon’s team at the department of polymer science and engineering, Kyungpook National University, who participated in the joint research. Covalent bonds are formed during the polymerization process between acrylamide and acrylic acid through the thermal radical formation of an initiator [19,20,21]. The polymerization process produces elastic and partially solid polymer chains as the final output. The polymer chains act as a bridge between the red clay particles after being mixed with the red clay binder, thereby filling the gaps among these particles. The solid entity of the PA solution, which encloses the silica particles of red clay and the moisture, immobilizes the stabilizer. The stabilizing process appears to improve the performance of the red clay binder (i.e., compressive strength, moisture resistance, and contaminant capture ability).

The poly(Acrylic acid(AA)-*co*-Acrylamide(AM)) is compounded by copolymerizing one monomer in the powder form (AM) and another monomer in the liquid form (AA) using an initiator. The AA-*co*-AM is then diluted with distilled water up to a certain concentration in which it is to be used as the PA solution. The solid polymer is converted into the liquid form to be mixed with water, an inexpensive solvent. Converting it to the powder form would have necessitated the use of costly organic compounds, such as toluene, as a solvent. In addition, the cost attributed to the manufacturing process and the number of additional processes would have increased, because additional solvents, such as ethanol and acetone, are required to remove the solvent attached to the fabricated polymer powder. This study investigates the curing characteristics of a red clay binder mixed with a PA solution, along with its compressive strength, water erosion, shrinkage deformation, and microstructure, thereby verifying its applicability to REC.

## 2. Experimental Procedures

### 2.1. Preliminary Experiment

**Figure 2:**
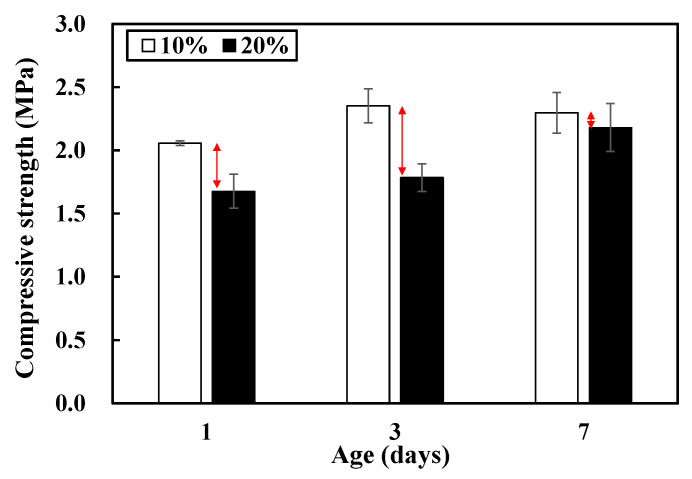
Compressive strength in different age and moisture content combinations.

A preliminary experiment was performed to determine the appropriate moisture content for the red clay binder and the optimal compressive load to be exerted on the specimen in the manufacturing process. A controlled experiment was conducted as follows. First, the specimen mixing option was set to RP (red clay + PA) to estimate the moisture content in the red clay binder. The compressive strength (i.e., an independent variable) was measured when the moisture content in the red clay sample (i.e., a dependent variable) was 10% and 20%. Figure 2 depicts the compressive strength of the red clay binder with different age and moisture content combinations. The compressive strengths of specimen that have 10% moisture content is higher than that of specimens that have 20% moisture content in all three ages (i.e., after curing for 1, 3, and 7 day(s)). It is worth noting that the compressive strength of a specimen that has 10% and 20% moisture content is 2.4 and 1.7 MPa, respectively, after curing for 3 days, indicating that the difference in the compressive strength is the greatest in the 3 day age. It is confirmed that 10% moisture content in the red clay sample leads to a higher strength development rate in the combination with the PA solution; hence, it is more favorable than the 20% moisture content.

**Figure 3:**
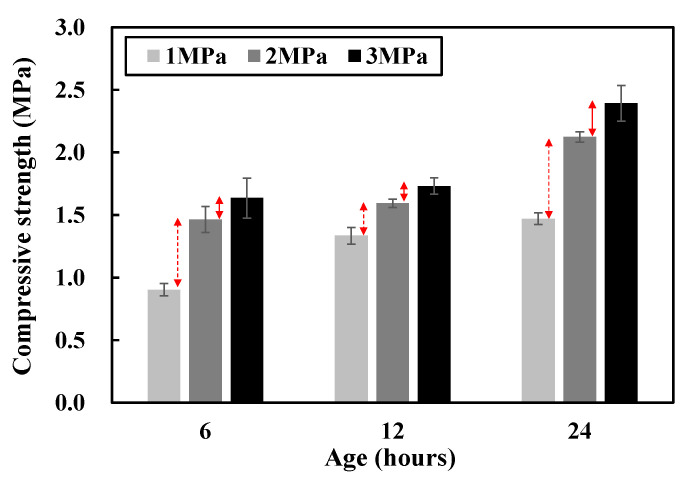
Compressive strength deviation in different compressive load and ages.

Second, the optimal compressive load is determined for specimens used in manufacturing the red clay binder. While the mixing combination of the specimen onto the RPC is kept constant (i.e., red clay + PA solution + cement), the compressive strength is measured by manipulating the compressive load with three loading values (i.e., 1, 2, and 3 MPa). Figure 3 shows the compressive strength measurement results for determining. The deviation between the compressive strength, given compressive loads of 1 MPa and 2 MPa, is approximately 0.3 MPa less, on average, than the deviation given those of 2 MPa and 3 MPa. It appears that the older the specimen age, the more the deviation. Given a compressive load of 1 MPa, the compressive strengths of the 6, 12, and 24 h specimens are 0.9, 1.3, and 1.5 MPa, respectively, which do not meet the optimal compressive strength requirement (i.e., 2 MPa) for REC (300 psi (2.07 MPa)) [6].

### 2.2. Core Experiment

#### 2.2.1. Materials

**Figure 4:**
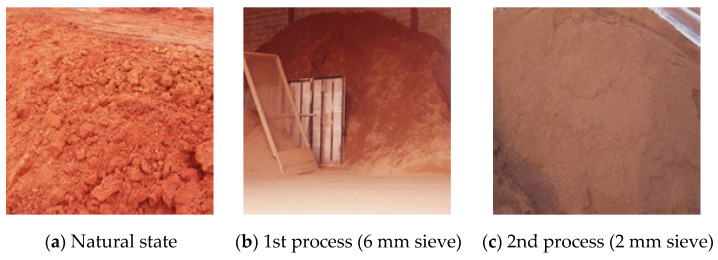
Red clay.

In the case of commercial red clay, it has a porous structure containing numerous halloysite-based micropores, consisting chemically of SiO_2_, Al_2_O_3_, Fe_2_O_3_, and its chemical composition is similar to other pozzolanic materials [22,23,24]. Figure 4 shows the red clay used in this study. To ensure a uniform particle size, the clay used in this study underwent natural drying after collection and was sifted through a 2 mm sieve before mixing. The density and loss ignition of the red clay was 2.7 g/cm³ and 9.27%, respectively.

The mass fraction and intrinsic viscosity of the PA solution, which is in the form of poly(AA-*co*-AM) and is obtained by copolymerizing AA (Acrylic acid; CHEMICAL DUKSAN, Incheon, Republic of Korea) and AM (Acrylamide; CHEMICAL DUKSAN, Incheon, Republic of Korea) using an initiator, are 5 wt% and a value between 2 and 3 dL/g, respectively [25]. In addition the Mw (molecular weight) and polydispersity of the PA solution are 54,000 g/mol and 2.46. The Type 1 ordinary Portland cement (OPC) of KS L 5201 used in the study has a density and fineness of 3.14 g/cm^3^ and 3500 cm^2^/g, respectively. The chemical compositions of the clay and cement used for this study are presented in Table 1.

#### 2.2.2. Experimental Plan

The manipulated and the controlled variables used in the experiment are presented in Table 2. The mixing ratios of the red clay, cement, and PA solution, which are independent variables, were manipulated to identify the curing characteristics, durability, and microstructure of the red clay binder. Three specimen types (i.e., R, RP, and RPC) were prepared as follows: R was the pure red clay; RP was obtained by adding the PA solution, a new additive, to red clay; and RPC was obtained by adding the PA solution and cement to red clay. Either the cement replaced 5 wt% of the binder and/or water or the PA solution, which amounts to 8 wt% of the binder, was added to the binder to meet the polymerization condition of 5 wt% poly(AA-*co*-AM). The threshold compressive load of 2 MPa, obtained through the preliminary experiment, was applied using a universal testing machine (UTM). The specimen was then cured under a constant temperature of 20 °C and a relative humidity of 60%. The hardening and deformation characteristics of the specimen were investigated by measuring the compressive strength, water erosion, and shrinkage. X-ray fluorescence (XRF) and X-ray diffraction (XRD) were performed for qualitative and quantitative analyses on the microstructures by using scanning electron microscopy (SEM) and mercury intrusion porosimetry (MIP).

#### 2.2.3. Experimental Method

##### Specimen Production

**Figure 5:**
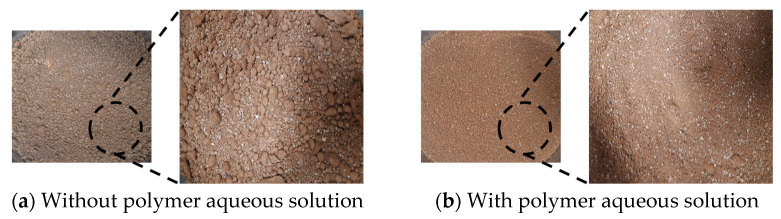
Surface changes of red clay binder with and without the polymer aqueous solution.

The red clay binder was mechanically mixed using a mixer. After weighing the binder sample, it was poured into a mixer container. Dry mixing was performed at the speed of (140 ± 5) rpm/min for 30 s. Then, the additive was distributed evenly in the mixer, and the mixer was operated at the same speed for an additional 1 min before stopping. The red clay binders that remained at the bottom and wall of the mixer container were detached and collected in the middle of the container, and the mixer was operated again at the same speed for another 1 min. The changes in the surface characteristics of the binder without the PA solution and after adding the PA solution are shown in Figure 5. The one suppresses the viscous force because the hydrogen bonds, acting as a bridge for the polymer, hold the silica and the water particles of the red clay together, thereby immobilizing the red clay binder. The hydrogen bonds may compromise the constructability involved in mixing and compacting red clay when this option is used. The other forms a set of aggregates owing to the viscous force generated by the large amount of CaCO_3_ existing in red clay during combination with water.

**Figure 6:**
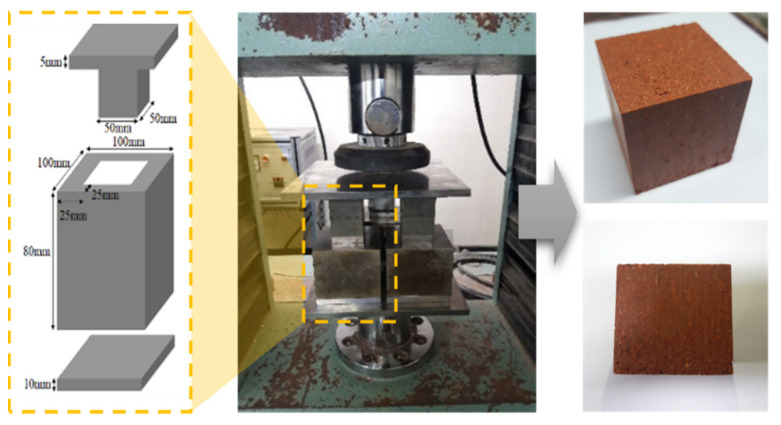
Mold, manufacturing apparatus, and specimen.

Specimens with dimensions of 50 mm × 50 mm × 50 mm were manufactured by using the mold and manufacturing apparatus shown in Figure 6. The mold was filled with a 240 g sample by pouring the materials three times in the same quantity, considering the gravity of the red clay and the threshold compressive load. Specimens were compacted uniformly by applying 2 MPa with the UTM. The fabricated specimens were cured in a chamber at a constant temperature and humidity as already mentioned.

##### Compressive Strength

The compressive strengths of the specimens at each age were measured as follows. Three specimens with the same dimensions (i.e., 50 mm × 50 mm × 50 mm) in each age (i.e., 1, 3, 7, and 28 days) were prepared in accordance with KS L 5105. The bearing loads on the specimens at each age were measured using the UTM. Subsequently, their compressive strengths were obtained by dividing the average bearing load by the area of the specimens in contact with the top mold cover.

##### Water Erosion

Water erosion was measured after curing the specimens for 7 days. The entire surface of some specimens were coated with inorganic ceramic resin (“CERAST” red clay waterproof coating), and some specimens were not coated. The other specimens were coated with two layers: the bottom layer with inorganic ceramic resin to impart permeation to the coating material, and the top layer was coated 30 min after coating the bottom layer. Each specimen was then immersed in water, and water erosion was observed every 3 h for 48 h.

##### Shrinkage

Shrinkage was measured after the specimens underwent curing in the controlled temperature and humidity (i.e., 20 °C and 60% RH) for 1 day. PS (TML; Tokyo Measuring Instruments, Tokyo, Japan) adhesive was coated on the surface of each specimen twice, thereby closing the pores on the surface of the specimens. Strain gauges, PFL-10-11-1LJC (TML, Japan), were then attached to both sides of each specimen using the cyanoacrylate (CN; Tokyo Measuring Instruments, Tokyo, Japan) adhesive. After connecting them to a data logger, TDS-303, the shrinkage was measured at 10 min intervals for 28 days.

##### X-ray Fluorescence

The XRF was investigated using a wavelength dispersive XRF spectrometer (WD-XRF; BRUKER; Seongnam, Republic of Korea) by complying with KS L 5120. The specimens were fabricated, and the samples were collected, converted to the powder form, and then preprocessed for measurement.

##### X-ray Diffraction

The XRD was measured using an X-ray diffractometer by complying with KS M 0043. The samples, which were collected from the specimens with ages of 7 and 28 days, were preprocessed. The 2-theta range was set to 5–80° for the measurement.

##### Scanning Electron Microscopy

Microstructures were observed using a scanning electron microscope (SNE-3200M; SEC, Suwon, Republic of Korea). The powdered samples collected at 7 and 28 days were coated with Pt. Then, they were observed under magnifications of 1000× and 3000×, respectively, at an acceleration voltage of 15 kV.

##### Mercury Intrusion Porosimetry

A porosity analyzer (AutoPore IV 9520; ZEUS, Daejeon, Republic of Korea) was used to investigate the microstructure porosity. The samples collected from the specimens at 7 and 28 days were completely dried using a drying furnace at 60 °C. Non-wetting mercury was pressurized from 0 to 60,000 psi. The porosity and pore size of the sample were measured by assessing the amount of mercury intrusion.

## 3. Results and Discussion

### 3.1. Compressive Strength

**Figure 7:**
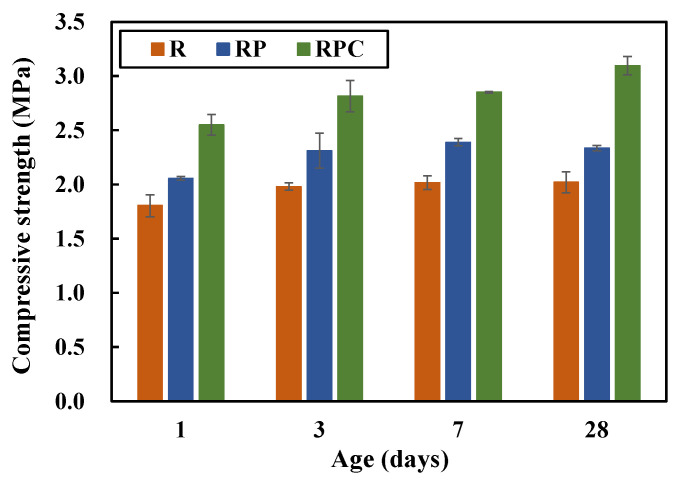
Compressive strengths of the four mixing combinations.

The compressive strengths of the red clay binder specimens are shown in Figure 7. RP exhibits slightly higher compressive strengths than R. It is confirmed that the solid poly(AA-*co*-AM) in the PA solution contributes to the improvement of the binder’s strength by serving as a secondary bond between the moisture molecules and the silica particles in red clay and forming a regular arrangement of particles. The performance of the PA solution may be improved by increasing the solid content of the PA solution while copolymerizing AA and AM.

RPC exhibits considerably higher compressive strengths than R and RP. It is remarkable that the compressive strength of RPC on the first day is 2.5 MPa, which meets the “28 days” compressive strength standard (2.4 MPa) recommended by ACP-EEC [5]. The strength improvement can be attributed to the secondary bonds of the PA solution, and the hydration reaction of cement contributes to the enhancement. Existing studies have claimed that the fit-for-use strength may be achieved by adding more than 8–12% of cement in REC [4,14]. It is found that RPC, which reduces the amount of cement added to 5 wt% of the binder, outperforms with respect to the compressive strength criteria suggested by the regulation.

### 3.2. Water Erosion

The water erosion involved in the addition and non-addition of the PA solution is presented in Table 3. In the specimens in which the surface was not coated, the bonds between the clay particles decomposed immediately after the specimens were saturated by water. Water erosion progressed rapidly for 3 h after water saturation for all four combinations, reaching complete erosion after 24 h. This confirmed that binder specimens that do not have surface coating with CERAST do not have sufficient water resistance. Indeed, the prerequisite for red clay binders for structural materials is the development of the counter measure of a surface waterproof coating material [26].

The coated red clay binder specimens exhibited water resistance for 48 h after being saturated by water. The R type specimen retained its structure for 3 h after being saturated by water. However, it experienced erosion by 24 h owing to water infiltration. As the R type specimen is composed of pure clay particles only, the water penetrates it through the micropores or microcracks on the binder surface, thereby weakening the chemical bonds and reducing its water resistance. For the RP and RPC type specimens with the PA solution, the PA solution improves the binding force between the clay particles owing to the hydrogen bonds of poly(AA-*co*-AM). Certainly, the addition of the PA solution increases the water resistance of the red clay binder by forming aggregates and retaining the shape of the specimens.

### 3.3. Shrinkage

The shrinkage of red clay may be larger than that of concrete because the clay has numerous porous molecular structures, resulting in higher water absorption than cement [27].

**Figure 8:**
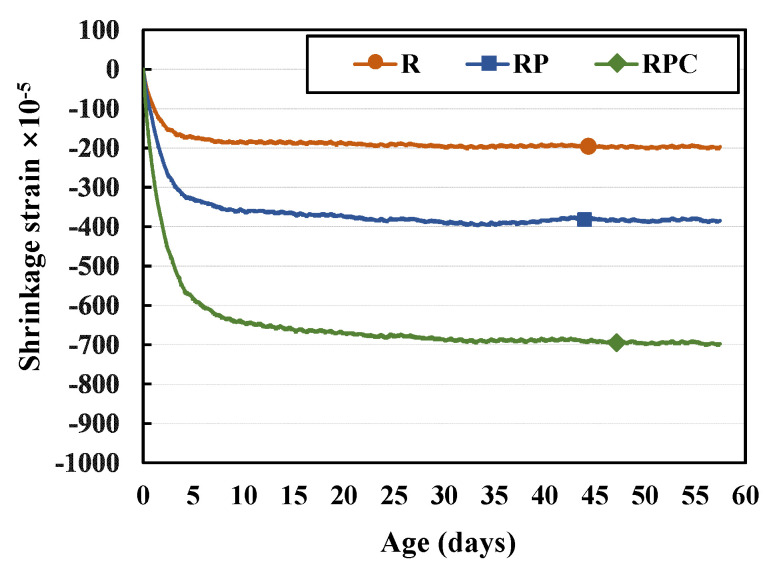
Shrinkage deformations occurring in specimens of different ages.

The shrinkage deformation occurs rapidly in all three red clay binder specimens (i.e., R, RP, and RPC) in their early ages, as shown in Figure 8. It decelerates gradually as their age increases. The shrinkage deformation of RP is larger than that of R. This can be explained by the evaporation of water molecules held together by hydrogen bonds between the solid poly(AA-*co*-AM) in the PA solution and the silica particles of red clay. The compressive strength of RP is slightly higher than that of R. It appears that the binding force attributed to the hydrogen bonds between poly(AA-*co*-AM) and silica particles contributes to the strength improvement. The shrinkage deformation of RPC is larger than that of R and RP in the early age. The volume change of the red clay binder, which occurred owing to the micropores generated inside the binder as the cement absorbs the moisture from the binder during the hydration reaction, may contribute to the deviation.

### 3.4. X-ray Fluorescence (XRF)

The main ingredients of R, RP, and RPC are SiO_2_, Al_2_O_3_, Fe_2_O_3_, and CaO, which can be confirmed through XRF analysis of the red clay binder, as shown in Table 4. The mechanical performance of each specimen varies based on the compositional ratio of CaO and SO_3_. It is well known that the compositional ratios of CaO and SO_3_ in OPC are approximately 66% and 2%, respectively [28,29]. The ratios of CaO and SO_3_ in the RPC are higher than those in R and RP. In addition, the ratios of SiO_2_ and Al_2_O_3_ in all red clay binder specimens vary from 75% to 81%. Certainly, the addition of moisture encourages strength development by catalyzing the pozzolanic reaction with Ca(OH)_2_.

### 3.5. X-ray Diffraction (XRD)

**Figure 9:**
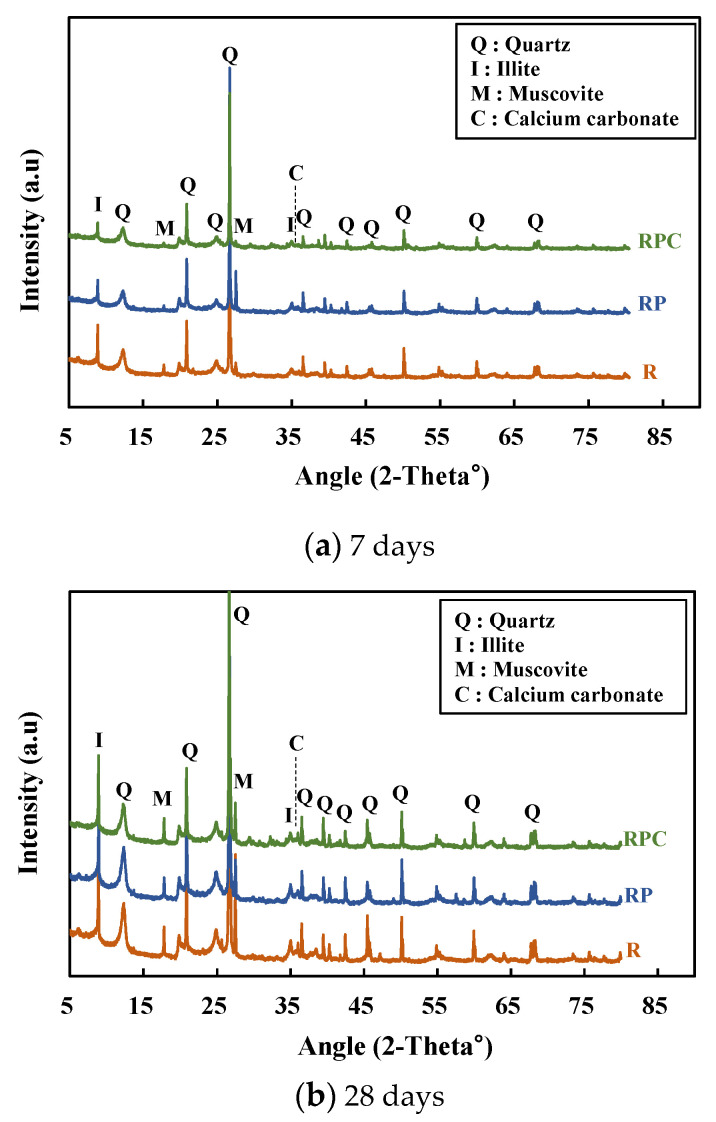
X-ray diffraction of specimens with the polymer aqueous solution.

XRD analysis on specimens with the ages of 7 and 28 days are shown in Figure 9. It was found that all three specimens (i.e., R, RP, and RPC) of the 7 days age have similar XRD patterns. Most of the major crystalline peaks in the analysis results were obtained from red clay [22,30], and most of the red clay binder specimens could be identified through the crystalline peaks corresponding to quartz, kaolinite, illite, and muscovite. The height of the quartz peak is very high within the 2-theta 5–80° range owing to the SiO_2_ occupying most of the red clay binder specimen volume. The variability of diffraction intensity is observed in each red clay binder specimen with a specific mixing combination type. This may be attributed to the different homogenization that occurred in the sample collection process. CaCO_3_, produced by the reaction between calcium oxide and water, was detected in the 2-theta 37° range. It should be noted that the calcium oxide was confirmed through the aforementioned XRF. These findings may contribute to the improvement of the binding force and the binder performance, in addition to the additives.

### 3.6. Scanning Electron Microscopy (SEM)

**Figure 10:**
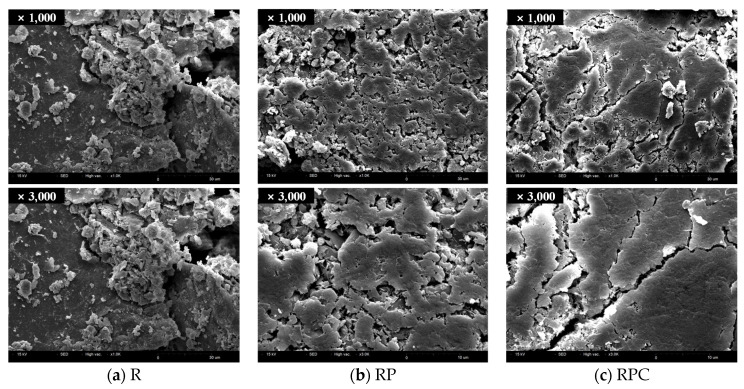
Scanning electron microscopy micrographs of specimens of 7 days age.

**Figure 11:**
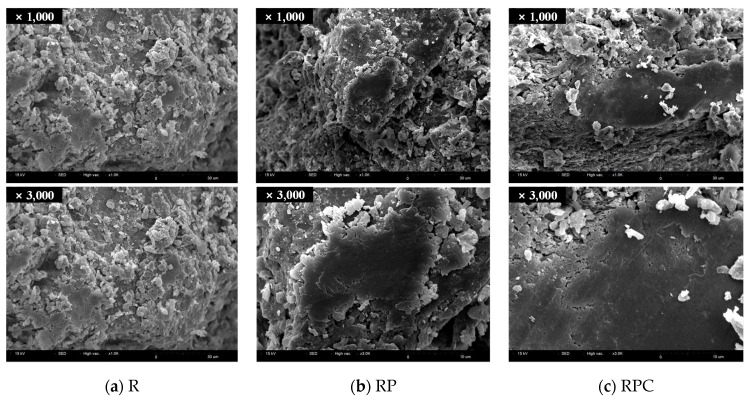
Scanning electron microscopy micrographs of specimens of 28 days age.

The SEM micrographs of red clay binder specimens of 7 days and 28 days ages are shown in Figure 10 and Figure 11, respectively. The surfaces of the R specimens were magnified to 1000× and 3000×, respectively. The surfaces of these specimens were considerably rough because the large and small silica particles were exposed. The insufficient aggregation of silt-sized particles, which dominate the main skeleton of the red clay binder, contributes to the roughness [31]. The fact that the average diameter of the silt occupying the specimen is 10 µm (0.01 mm) proves that the specimen is mainly composed of silt, whose standard diameter is between 0.002 to 0.074 mm.

Conversely, the surface of RP and RPC are smoother than those of R as the poly(AA-*co*-AM) acts as a bridge for secondary bonds by creating effective hydrogen bonds with the silica particles and moisture molecules. The plate-like surface observed in the 28 day age confirms that the silica particles of RPC in the 28 day age forms stronger aggregates than those in the 7 day age.

### 3.7. Mercury Intrusion Porosimetry (MIP)

**Figure 12:**
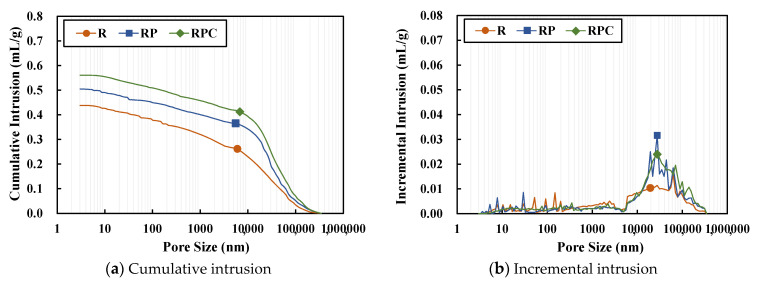
Mercury intrusion porosimetry outputs of the specimens of 7 days age.

**Figure 13:**
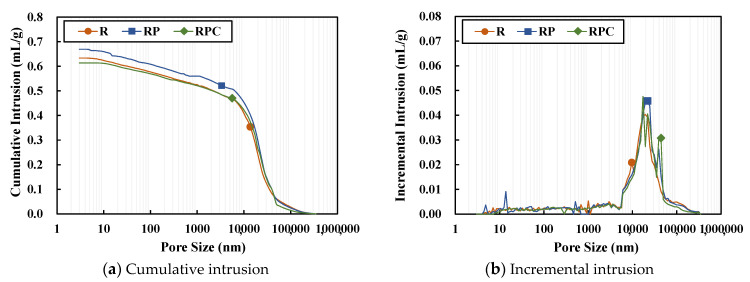
Mercury intrusion porosimetry outputs of the specimens of 28 days age.

The MIP analysis on the red clay binder specimens of 7 days and 28 days age confirms that diameter of the pores inside the red clay binder specimens is between 10^4^ and 10^5^ nm, as shown in Figure 12 and Figure 13, respectively. The cumulative porosities of RP and RPC are higher than those of R for the 7 days age. This may be because of the copolymerization of AA and AM using the initiator, which formulates the poly(AA-*co*-AM). It appears that the molecules are at a distance of approximately 10^4^–10^5^ nm from each other during the molecular bonding in the copolymerization process, thereby affecting the cumulative porosities [19]. Although the cumulative porosity of RP is higher than that of R, the compressive strength of RP is higher than that of R. It appears that the strong binding force achieved by the hydrogen bonding between the solid polymer and the silica particles contributes to the strength improvement in RP.

After 28 day, the cumulative porosity of all four types of red clay binder specimens increased owing to the volumetric increase in pores caused by the shrinkage as they aged. Meanwhile, for RPC, the cumulative porosity was measured at a lower level than for RP and R. It is judged that hydration products, such as calcium hydroxide, produced by the hydration reaction of cement filled the pores, and that the secondary combination of poly(AA-*co*-AM) resulted in a higher compressive strength than other specimens.

## 4. Conclusions

This study confirms the performance (i.e., compressive strength, water erosion, shrinkage, and microstructure) of each red clay binder specimen by manipulating the independent variables (i.e., the mixing ratios of red clay, cement, and PA solution). 

The findings are as follows. The compressive strength of the RP type specimen is higher than that of the R type specimen. The poly(AA-*co*-AM), which includes polymer solid content within 5 wt% in the PA solution, forms a regular arrangement between the moisture molecules and the silica particles, contributing to the compressive strength improvement. The red clay binder specimens that do not have surface coating do not exhibit water resistance. The specimens with surfaces enclosed with a water-resistant coating material retain the resistance to water erosion. It would be desirable to implement a counter measure to ensure the water resistance of the binder when making use of the red clay binder for practical applications.

Furthermore, the shrinkage deformation of RP is larger than that of R in the early ages. However, the difference between the two does not increase with the age. Indeed, the measure to control shrinkage crack can be recommended as future research. The microstructural observation of the RP and RPC specimens confirms the plate-like surfaces, thereby proving that the silica particles of clay create more efficient hydrogen bonds owing to the polymer solid poly(AA-*co*-AM) within the PA solution. The two specimens with PA solution create more effective aggregates than the two specimens without PA solution. In addition, the cumulative porosities of the specimens with PA solution are higher than those of specimens without PA solution as the molecular distance between the moisture molecules and/or the silica particles increase in the copolymerization process. However, despite the cumulative porosities in the RP and RPC specimens, their compressive strengths significantly increase owing to the addition of PA solution to the red clay binder, which improves the compressive strength and durability because the poly(AA-*co*-AM) effectively enhances the hydrogen bonds with the silica particles within clay.

In conclusion, the addition of the PA solution to the red clay binder contributes to the improvement of the strength and durability of the binder owing to the effective hydrogen bonding of poly(AA-*co*-AM) with the silica particles of red clay. Although the addition of the PA solution marginally increases the porosity and shrinkage of the binder, wide practical applicability of the solution as a new additive is expected because it meets the construction criteria while reducing the amount of cement used in REC.

## Figures and Tables

**Table 1:** Chemical compositions of materials.

Materials	Chemical Composition (%)										
	SiO_2_	Al_2_O_3_	K_2_O	Fe_2_O_3_	TiO_2_	MgO	CaO	Pd	Ru	ZrO_2_	LOI
Red clay	56.79	24.87	4.63	3.99	0.74	0.62	0.13	0.07	0.06	0.04	7.95
OPC	20.70	6.20	0.84	3.10	-	2.80	62.20	-	-	-	1.96

**Table 2:** Experimental plan.

Type	Manipulated Variables				Controlled Variables		Parameters for Evaluation
	Binder (wt%)		W (B × wt%)	PA (B × wt%)	Consolidation Condition	Curing Conditions	
	R	C					
R	100	-	8	-	2 MPa	20 ℃ RH 60%	Compressive strength Water erosion Shrinkage XRF XRD SEM MIP
RP	100	-	-	8			
RPC	95	5	-	8			

Note: R: red clay, C: cement, W: mixing water, PA: polymer aqueous solution; Note: P(polymer) 5% [inner part] = PA × wt%.

**Table 3:** Water erosion of the red clay binder specimens.

Without Coating					With Coating				
Spec.	After 0 h	After 3 h	After 24 h	After 48 h	Spec.	After 0 h	After 3 h	After 24 h	After 48 h
R	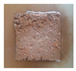	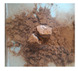	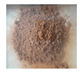	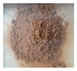	R	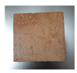	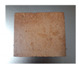	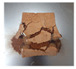	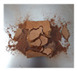
RP	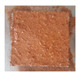	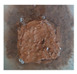	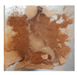	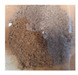	RP	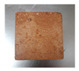	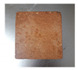	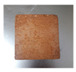	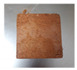
RPC	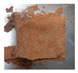	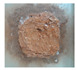	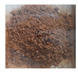	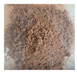	RPC	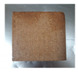	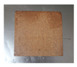	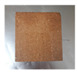	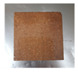

**Table 4:** X-ray fluorescence analysis after adding the polymer aqueous solution.

Specimen Types.	Chemical Composition (%)													
	SiO_2_	Al_2_O_3_	Fe_2_O_3_	K_2_O	TiO_2_	MgO	CaO	Cl	CuO	SO_3_	Ru	MnO	Na_2_O	LOI
R	59.16	22.38	4.69	2.84	0.89	0.64	0.19	0.15	-	-	0.07	0.05	-	8.89
RP	58.22	22.74	4.78	3.03	0.88	0.61	0.15	-	0.03	-	-	-	-	9.38
RPC	55.31	19.98	4.36	2.60	0.88	0.85	5.37	-	0.02	0.19	-	0.04	-	10.2
